# Genetic characteristics of *Giardia duodenalis* from sheep in Inner Mongolia, China

**DOI:** 10.1051/parasite/2020060

**Published:** 2020-11-16

**Authors:** Letian Cao, Kelei Han, Luyang Wang, Surong Hasi, Fuchang Yu, Zhaohui Cui, Ying Hai, Xinguo Zhai, Longxian Zhang

**Affiliations:** 1 College of Animal Science and Veterinary Medicine, Henan Agricultural University 450046 Zhengzhou PR China; 2 International Joint Research Laboratory for Zoonotic Diseases of Henan 450046 Zhengzhou PR China; 3 Inner Mongolia Agricultural University 010018 Hohhot PR China; 4 Wushen Banner Center for Animal Disease Control and Prevention 017300 Ordos PR China; 5 Zhengzhou Municipal Agriculture Rural Work Committee 450006 Zhengzhou PR China

**Keywords:** Inner Mongolia, Sheep, *Giardia duodenalis*

## Abstract

*Giardia duodenalis* is an important zoonotic pathogen for both human and animal health. Although there have been reports on *G. duodenalis* infections in animals all over the world, information regarding the prevalence and genetic characteristics of *G. duodenalis* in sheep in Inner Mongolia, China, is limited. In this study, 209 sheep fecal specimens were collected in this autonomous region. We established that the prevalence of *G. duodenalis* was 64.11% (134/209), as determined using nested PCR detection and sequences analysis of the small subunit ribosomal RNA (*SSU rRNA*) gene. Based on the beta-giardin (*bg*) locus, the glutamate dehydrogenase (*gdh*) locus, and the triose phosphate isomerase (*tpi*) locus to study genetic characteristics, both assemblages A (2.99%, 4/134) and E (97.01%, 130/134) were found. Five novel nucleotide sequence of assemblage E were detected, two at the *bg* locus, two at the *gdh* locus, and one at the *tpi* locus. Multilocus genotyping yielded four assemblage E and two assemblage A multilocus genotypes (MLGs), including four novel assemblage E MLGs and one novel assemblage A MLG. Results of this study indicated that *G. duodenalis* was highly prevalent in sheep in Inner Mongolia. This study is the first to use the multilocus genotyping approach to identify *G. duodenalis* in sheep from this region.

## Abstract (fr)

*Giardia duodenalis* est un pathogène zoonotique important pour la santé humaine et animale. Bien qu’il y ait eu des rapports sur des infections à *G. duodenalis* chez les animaux partout dans le monde, les informations concernant la prévalence et les caractéristiques génétiques de *G. duodenalis* chez les ovins en Mongolie intérieure, Chine, sont limitées. Dans cette étude, 209 spécimens fécaux de moutons ont été collectés dans cette région autonome. Nous avons établi que la prévalence de *G. duodenalis* était de 64,11 % (134/209), grâce à la détection par PCR imbriquée et l’analyse des séquences de la petite sous-unité du gène de l’ARN ribosomal (ARNr SSU). Sur la base du locus bêta-giardine (*bg*), du locus glutamate déshydrogénase (*gdh*) et du locus triose phosphate isomérase (*tpi*) pour l’étude des caractéristiques génétiques, les deux assemblages A (2,99 %, 4/134) et E (97,01 %, 130/134) ont été trouvés. Cinq nouvelles séquences nucléotidiques de l’assemblage E ont été détectées, deux au locus *bg*, deux au locus *gdh* et une au locus *tpi*. Le génotypage multilocus a produit quatre génotypes multilocus (GML) pour l’assemblage E et deux GML pour l’assemblage A, y compris quatre nouveaux GML pour E et un nouveau GML pour A. Les résultats de cette étude indiquent que *G. duodenalis* était très répandu chez les moutons en Mongolie intérieure. Cette étude est la première à utiliser l’approche de génotypage multilocus pour identifier *G. duodenalis* chez les moutons de cette région.

## Introduction

*Giardia duodenalis* (also known as *G. lamblia* or *G. intestinalis*) is a common intestinal parasite that is widespread among vertebrate hosts, including humans, livestock, and wildlife, worldwide [8, 34]. *Giardia duodenalis* infections often remain asymptomatic, but can cause severe diarrhea and chronic disease in humans [6, 16, 24]. Investigations and case reports on *G. duodenalis* infections in humans are common in China. The large number of epidemiological investigations conducted at the start of this century suggested that the average infection rate was 0.85% (197/23,098) [18], with the highest infection rate (9.46%, 7/74) reported by one study carried out in a pediatric hospital in China [32]. As sheep have been found to have unexpectedly high levels of infection, they have long been considered a potential reservoir for human infections [10, 25, 31].

Extensive analysis of protein and DNA polymorphisms have long been considered findings indicating that *G. duodenalis* is a species complex, whose members show little variation in their morphology, and the major genetic groups are now described as assemblages (may correspond to distinct species) [30]. Studies have shown that *G. duodenalis* can be sub-classified into at least 8 genetically different assemblages (A–H) [29], of which assemblage A and assemblage B are considered to be zoonotic, while the remaining assemblages (C–H) seem to be host-specific. However, in recent studies, assemblage C, D, E and F has been found in a few human cases [1, 7, 26, 37]. Studies on sheep have identified a predominance of *G. duodenalis* assemblage E, while assemblage A occurred infrequently [10, 25, 27, 31] and assemblage B was rarely found [5, 23].

For the past few years, the reported infection rate with *G. duodenalis* in sheep and goats in China was 6.07% (418/6890) [18]. Among these, almost all the cases of *G. duodenalis* infections in sheep were caused by assemblages E and A, with assemblage E being particularly prevalent. However, there are few reports on *G. duodenalis* infection rates in goats and sheep in Inner Mongolia [36, 40].

In recent years, multilocus genotyping (MLG) of the beta-giardin (*bg*), glutamate dehydrogenase (*gdh*) and triose phosphate isomerase (*tpi*) loci has increasingly been used to characterize *G. duodenalis* infections in humans and animals [4, 11, 33]. This method has been favored because PCR assays targeting these loci have been shown to have different sensitivities, and occasionally different genotyping results [19, 22]. However, most earlier studies characterized *G. duodenalis* in sheep using individual loci, and thus far, there are only a few reports on genotyping *G. duodenalis* from sheep in China using MLG analysis [39].

The Ordos fine-wool sheep is a unique breeding animal in Wushen Banner, Ordos City, Inner Mongolia Autonomous Region. Its fur and meat have extremely high economic benefits and are the main local economic animals, playing an important role in animal husbandry in this area. *Giardia duodenalis* has influence on the growth and development of sheep, which may in turn affect the economic benefits for local farmers [2]. However, data on *G. duodenalis* infection in Ordos fine-wool sheep are rare.

The objectives of this study were to investigate the distribution of *G. duodenalis* assemblages/genotypes in sheep in Inner Mongolia based on MLG analysis, and analyze their genetic characteristics, assess the zoonotic transmission risk, and elucidate the public health significance of this protozoan parasite.

## Materials and methods

### Ethics statement

This study was performed in accordance with the recommendations of the Guide for the Care and Use of Laboratory Animals (Publication Year: 2010, ISBN: 9780309154000). The research protocol was reviewed and approved by the Research Ethics Committee of Henan Agricultural University (approval no. LVRIAEC 2018-007). Permission was obtained from the farm owners before fecal sample collection. In this study, all fecal samples were carefully collected from the rectum of each sheep without causing discomfort.

### Sample collection

The Inner Mongolia Autonomous Region straddles three major regions of northeast China, north China and northwest China. The area has a plateau-type geology, with a complex and diverse temperate continental monsoon climate. Inner Mongolia makes good use of its local geographical and meteorological features, and is the largest grassland pastoral area in China, with animal husbandry making an important economic contribution.

To study the infection rate and aggregation distribution of *G. duodenalis* in this animal population, we selected the representative commercial farm at our study site that has the highest intensity of Ordos fine-wool sheep (>3 months old). There were no symptoms of diarrhea in the flock during sample collection. Fresh feces were collected from animals by rectal sampling and stored in a 2.5% (w/v) potassium dichromate solution in clean containers. Stool samples were of normal shape. All fecal specimens were transported to the laboratory with an ice pack at 4 °C immediately after collection. DNA extraction was performed within 48 h.

### DNA extraction and PCR amplification

DNA extraction was performed using commercial E.Z.N.A Stool DNA kits (Omega Bio-Tek Inc., Norcross, GA, USA), following the manufacturer’s recommendations. Extracted DNA samples were stored at −20 °C until PCR analysis.

The DNA samples were analyzed using nested PCR amplification of the small subunit ribosomal RNA (*SSU rRNA*) gene to determine the *G. duodenalis* infection rate [3]. Additionally, to determine the multilocus genotypes (MLGs) of the *G. duodenalis* isolates detected in this study, all *G. duodenalis* positive isolates were tested using nested PCR based on the *bg* [15], *gdh* [4] and *tpi* [28] loci (Table 1). Using an Applied Biosystems 2720 Thermal Cycler (Applied Biosystems, Foster City, CA, USA), PCR reactions for *G. duodenalis* loci were conducted in 25 μL systems: 2.5 μL 10× PCR buffer, 2 μL dNTPs (1.25 mM each), 0.3 μL each primer (25 μM each), 0.2 μL rTaq DNA polymerase (1 unit/μL each) (Takara Shuzo Co., Ltd), 2 μL of DNA sample, 17.7 μL double distilled water.

**Table 1:** Primer sequences and reaction conditions used in nested PCR amplifications.

Gene	Primer sequences (5′ – 3′)	Nucleotide fragment (bp)	Annealing temperature (°C)	Reference
*SSU rRNA*	Gia2029 (AAGTGTGGTGCAGACGGACTC)	292	55	[3]
	Gia2150c (CTGCTGCCGTCCTTGGATGT)			
	RH11 (CATCCGGTCGATCCTGCC)		59	
	RH4 (AGTCGAACCCTGATTCTCCGCCCAGG)			
*bg*	BG1(AAGCCCGACGACCTCACCCGCAGTGC)	511	65	[15]
	BG2(GAGGCCGCCCTGGATCTTCGAGACGAC)			
	BG3 (GAACGAACGAGATCGAGGTCCG)		55	
	BG4 (CTCGACGAGCTTCGTGTT)			
*gdh*	Gdh1 (TTCCGTRTYCAGTACAACTC)	520	50	[4]
	Gdh2 (ACCTCGTTCTGRGTGGCGCA)			
	Gdh3 (ATGACYGAGCTYCAGAGGCACGT)		50	
	Gdh4 (GTGGCGCARGGCATGATGCA)			
*tpi*	AL3543 (AAATIATGCCTGCTCGTCG)	530	50	[28]
	AL3546 (CAAACCTTITCCGCAAACC)			
	AL3544 (CCCTTCATCGGIGGTAACTT)		50	
	AL3545 (GTGGCCACCACICCCGTGCC)			

The secondary PCR products were separated by 1% agarose gel electrophoresis, following staining with DNA Green (TIANDZ, Beijing, China), observed, photographed, and recorded on a Tanon 3500 Gel Image Analysis System (TANON, Shanghai, China).

### Sequence and phylogenetic analyses

All the secondary PCR amplicons of the *SSU rRNA*, *bg*, *gdh* and *tpi* genes from *G. duodenalis*-positive samples were bidirectionally sequenced using an ABI PRISM 3730 XL DNA analyzer with the BigDye Terminator v3.1 Cycle Sequencing Kit (Applied Biosystems, Foster City, CA, USA), owned by the Tsingke Biological Technology Co. Ltd (Beijing, China), and no double peaks were detected during chromatogram inspection.

The sequences obtained were assembled using ChomasPro 2.64 (http://www.technelysium.com.au), and edited using DNAstar Lasergene Editseq 7.1.0 (http://www.dnastar.com/). The upstream and downstream sequencing results are spliced into a consensus sequence, and the obtained genetic variants were analyzed by multiple-sequence alignments with reference sequences downloaded from the GenBank database, using Clustal X 2.1 (http://www.clustal.org/).

Positive samples at the *SSU* rRNA locus were analyzed at three other loci (*bg*, *gdh*, and *tpi*) to understand the genetic characteristics of Giardia; the MLGs of *G. duodenalis* were also identified using the sequence data of these loci. Sequences from each isolate at the three analyzed loci were concatenated (*bg*-*tpi*-*gdh*) to form one multilocus sequence for each isolate.

Neighbor-joining (NJ) analysis was performed using MEGA 7.0 software (http://www.megasoftware.net/), based on the Kimura-2 parameter model.

### Statistical analysis

The infection rates and 95% confidence intervals (CI) were calculated by the Wald method in SPSS, version 22.0 (SPSS Inc., Chicago, IL, United States). Differences in corresponding infection rates among locations were examined by the Chi-square test, and differences were considered significant at *p* < 0.05.

### Nucleotide sequence accession numbers

The representative nucleotide sequences generated in this study were submitted to the GenBank database under the accession numbers MK442896–MK442915.

## Results

### *Giardia duodenalis* prevalence, and distribution of assemblages

A total of 134 (64.11%, 95% CI: 57.6–70.7%) *G. duodenalis*-positive fecal samples were identified using the nested PCR analysis of the *SSU rRNA* genes in this study. The genetic diversity of the *G. duodenalis*-positive samples was determined by sequencing the *bg*, *gdh* and *tpi* genes, and a total of 39, 72 and 32 sequences, respectively, were obtained for these three genetic loci. Assemblage E (*n* = 130) and assemblage A (*n* = 4), were detected, based on the *SSU rRNA* gene.

### Assemblage A and E

Of the *bg* sequences, 7 were identified as assemblage A, and 32 were identified as assemblage E. Sequence A1 (*n* = 4) was identical to AY655702, and A2 (*n* = 3) had one single-nucleotide polymorphism (SNP) relative to AY072723 (Table 2). Assemblage E sequences were designated as E1 (*n* = 11), E2 (*n* = 12), E3 (*n* = 7), E4 (*n* = 1), and E5 (*n* = 1). The E3 and E4 had one SNP each (A170G and C428T) compared to KT922250 and KT922248, respectively and one sequence each was identical to MK610388, KT922250, and KP635098.

**Table 2:** Intra-assemblage substitutions in the beta-giardin (*bg*), glutamate dehydrogenase (*gdh*), and triose phosphate isomerase (*tpi*) assemblage A gene sequences.

Sequence (no.)	Nucleotide positions						GenBank ID
*bg*	87	336					
Ref. sequence	T	T					AY655702
A1(4)	–	–					MK442896
A2(3)	C	C					MK442897
*gdh*	1–514						
Ref. sequence	–	–	–	–	–		AY178735
A1(8)	–	–	–	–	–		MK442903
*tpi*	39	53	100	114	222	363	
Ref. sequence	C	A	A	C	G	C	EU041754
A1(3)	T	G	G	T	A	–	MK442911
A3(9)	–	–	G	–	–	T	MK442910

–: indicates that the sequence is the same as the reference sequence.

At *gdh* sequences, 8 were identified to assemblage A, and 64 were identified to assemblage E. All the 8 assemblage A sequences were identical to the genotype A1 sequence (AY178735) (Table 2). Among the assemblage E isolates, E3 and E6 had one SNP each (G369A and A455G) relative to MK645797 and MK645792, respectively. The remaining sequences were identical to counterparts in the database (E1, E2, E4 and E5 were identical to KT369778, KT369785, KY432862, and MK645788, respectively). Using KT369778 as the reference sequence, the intra-assemblage substitutions in assemblage E at the *gdh* gene can be seen in Table 3.

**Table 3:** Intra-assemblage substitutions in the beta-giardin (*bg*), glutamate dehydrogenase (*gdh*), and triose phosphate isomerase (*tpi*) assemblage E gene sequences.

Sequence (no.)	Nucleotide positions					GenBank ID
*bg*	65	170	383	413	428	
Ref. sequence	C	A	C	T	C	KT922248
E1 (11)	–	–	–	C	–	MK442898
E2 (12)	–	–	–	–	–	MK442899
E3 (7)^a^	–	G	–	–	–	MK442900
E4 (1)^a^	–	–	–	–	T	MK442901
E5 (1)	T	–	T	C	–	MK442902
*gdh*	131	272	359	445		
Ref. sequence	A	T	G	G		KT369778
E1 (26)	–	–	–	–		MK442904
E2 (14)	–	G	–	–		MK442905
E3 (9)^a^	–	–	A	–		MK442906
E4 (12)	–	G	–	A		MK442907
E5 (2)	G	–	–	–		MK442908
E6 (1)^a^	G	G	–	–		
*tpi*	29	87	137	308		
Ref. sequence	G	G	A	T		KT369763
E1 (10)	–	–	–	–		MK442912
E2 (5)	A	–	G	–		MK442913
E3 (4)	–	A	–	C		MK442914
E4 (1)^a^	–	–	–	C		MK442915

–: indicates that the sequence is the same as the reference sequence.

a Novel sequence.

Sequence analysis of the *tpi* locus revealed that 12 successfully amplified isolates were identified as assemblage A, and 20 were assemblage E. A1 was identical to L02120, and A3 had two SNPs (A100G and C363T) relative to EU041754. Among the assemblage E sequences, E4 was identified to be a novel sequence, and the remaining sequences were consistent with KT369763, KT922262, and MF671903, respectively. The intra-assemblage substitutions in assemblage E at the *tpi* gene can be seen in Table 3.

### Multilocus genotyping

Using multilocus sequence typing, 4 assemblage A and 5 assemblage E isolates were successfully sequenced at all three loci (Table 4). To study the relationships between the different isolates in more detail, we performed a phylogenetic analysis based on a dataset of concatenated *bg* + *gdh* + *tpi* gene sequences. Data from the specimens were not included in the MLG analysis when a mixed infection was detected at one of the three loci.

**Table 4:** Multilocus characterization of *Giardia duodenalis* isolates based on the beta-giardin (*bg*), glutamate dehydrogenase (*gdh*) and triose phosphate isomerase (*tpi*) genes.

Isolate	Genotype			MLG type
	*bg*	*gdh*	*tpi*	
1, 5, 9	A1	A1	A1	AI-1(IM)
16	E2	E4	E3	MLGE1(IM)
51, 54	E2	E3^a^	–	
52, 56, 64	–	E3^a^	–	
53	A1	A1	A3	AI-novel1(IM)
57	–	E3^a^	A3	
60	–	E3^a^	E4^a^	
61	A2	E3^a^	–	
70, 74	E1	E1	E1	MLGE2(IM)
72, 131	E3^a^	–	–	
122, 124, 126, 128	E3^a^	E1	–	
130	E3^a^	E1	E1	MLGE3(IM)
174	E2	E2	E2	MLGE4(IM)
202	–	E6^a^	–	
209	E4^a^	E2	A3	Mixed

–: indicates PCR negative isolates.

a Novel sequence.

IM: Inner Mongolia.

**Figure 1:**
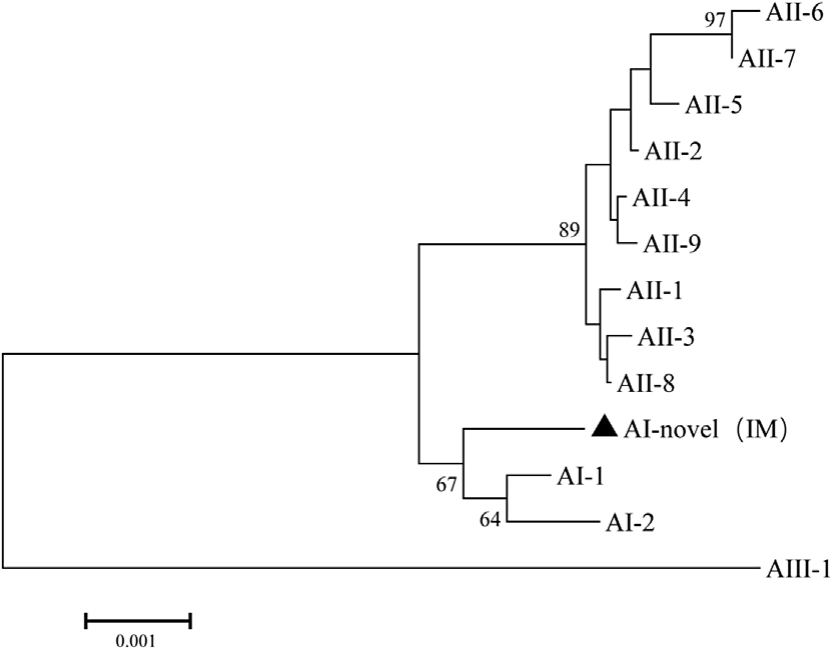
Phylogenetic relationships among *Giardia duodenalis* multilocus genotypes of sub-assemblage A. The filled triangles represent the isolates from Inner Mongolia Autonomous Region. The neighbor-joining tree was constructed using concatenated sequences of the beta-giardin (*bg*), glutamate dehydrogenase (*gdh*), and triose phosphate isomerase (*tpi*) genes, based on genetic distances calculated using the Kimura-2 parameter model.

**Figure 2:**
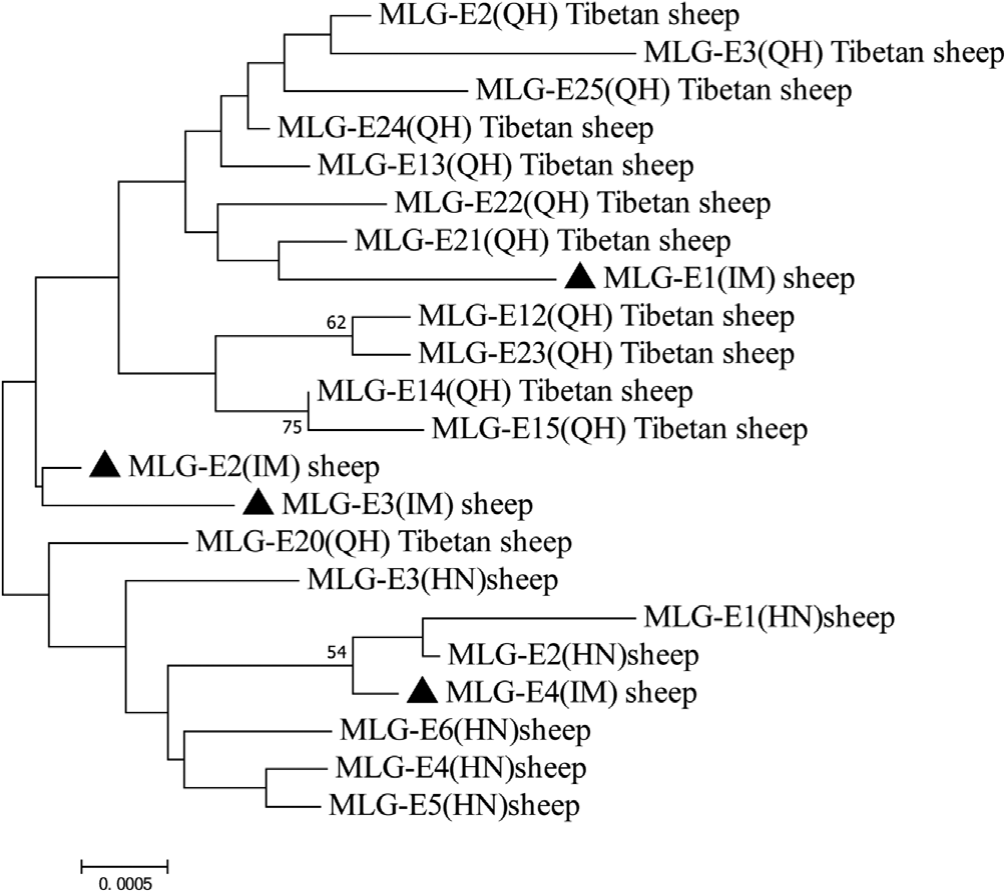
Phylogenetic relationships among *Giardia duodenalis* multilocus genotypes from sheep in China. The filled triangles represent the isolates from Inner Mongolia Autonomous Region. The neighbor-joining tree was constructed using concatenated sequences of the beta-giardin (*bg*), glutamate dehydrogenase (*gdh*), and triose phosphate isomerase (*tpi*) genes, based on genetic distances calculated using the Kimura-2 parameter model. QH: Qinghai; HN: Henan; IM: Inner Mongolia.

Multilocus genotyping yielded two assemblages A MLGs and four assemblage E MLGs. One assemblage A MLG was identical to the AI-1, and the assemblage A MLG was considered a novel MLG (named AI-novel (IM)) which had genetic distance with AI-1 and AI-2; AI-novel (IM) and AI were in the same cluster in the phylogenetic analysis (Fig. 1). The MLG-E2 and MLG-E3 from Inner Mongolia (IM) found in this study were genetically distinct from those found in sheep from other areas in China (Fig. 2).

## Discussion

*Giardia duodenalis* is an important intestinal parasite that has a global distribution in humans and a diverse range of other animals [8]. There have been reports of *G. duodenalis* infection in sheep in various regions of China, including Heilongjiang [20, 38], Henan [17], Jilin, Liaoning, Shandong [17], and Qinghai [21]. However, data on *G. duodenali*s infections in sheep in Inner Mongolia are limited, with only one published report [36]. This study reports on the occurrence and genetic characteristics of *G. duodenalis* infections in sheep in Inner Mongolia, China.

The results of this study showed that the occurrence rate of *G. duodenalis* in sheep was 64.11%, which is considerably higher than previously reported for sheep in Inner Mongolia (4.27%, 16/375) [36]. It is also higher than the infection rates of *G. duodenalis* reported for sheep from other regions of China, such as Heilongjiang (4.64%, 25/539) [38], Henan (5.24%, 100/1906) [17, 33], Jilin (0%, 0/48), Liaoning (0%, 0/16), Shandong (0%, 0/17) [17], Qinghai–Tibetan Plateau Area (0%, 0/65) [13], and Qinghai (13.11%, 8/61) [21, 40]. These differences in reported occurrence rates may be due to the livestock farming methods used (free-range or intensive farming), the age and health status of the animals, or the climate, as well as the sample size and detection methods used in the different studies [14].

In previous studies, apart from one study in which two assemblage B isolates were identified in sheep from Heilongjiang province [38], *G. duodenalis* infections in Chinese sheep were all reported to be caused by either assemblage E or assemblage A [18], which is consistent with the results of this study. Assemblage E is apparently the most common *G. duodenalis* genotype in sheep [8]. In this study, assemblage E accounted for 97.01% in sheep infected with *G. duodenalis*, which is also consistent with previous reports [33, 38]. Assemblage E is commonly found in hoofed animals, including sheep, and is not considered anthroponotic. However, several human cases have been reported in Egypt, Brazil and Australia [1, 7, 9, 12, 37], and additional research is therefore needed to study the public health risks of assemblage E.

Four assemblage E MLG genotypes were identified in total, all of which were new assemblage E MLG genotypes, indicating that assemblage E had high genetic diversity. The phylogenetic analysis of the concatenated sequences of assemblage E MLGs revealed that assemblage MLG-E2 (IM) and assemblage MLG-E3 (IM) found in this study were genetically distinct from the assemblages found in sheep in Qinghai and Henan Provinces, China [14, 33]. These differences were mainly due to the genetic variation of the *bg* locus. MLG-E1 (IM) were placed in the major cluster of MLGs from Tibetan sheep in Qinghai, whereas MLG-E4 (IM) clustered with MLGs from sheep in Henan Province (Fig. 1).

The phylogenetic analysis of the concatenated sequences of the assemblage A MLGs revealed that AI-novel (IM) was a new MLG that belonged to sub-assemblage AI. Sequences obtained from the assemblage A MLG isolates belonged to the sub-assemblage AI, which has been more commonly identified in animals than humans [8, 35]. Although *G. duodenalis* found in this study have limited zoonotic potential, a threat to public health cannot be ignored.

These MLGs results suggest that there was no significant geographic isolation of *G. duodenalis* genotypes in three regions in China. This may be because the Inner Mongolia Autonomous Region is the largest grassland pastoral area in China, and sheep fed here will be distributed to various regions of the country, thus promoting gene exchange of *G. duodenalis* in various regions. However, there may be specific genotypes in different regions for different breeding environments, and this still needs to be investigated in extensive further research.

In conclusion, the results of this study showed that there was a high prevalence of *G. duodenalis* in sheep from Inner Mongolia, in northwest China. Both assemblages A and E were found, with assemblage E being the most prevalent type. Two new *bg* gene sequences, two new *gdh* gene sequences, and one new *tpi* gene sequence was identified. Multilocus genotyping yielded four new assemblage E MLGs and one new sub-assemblage A MLG. In addition, further studies on the zoonotic potential and geographic isolation of *G. duodenalis* from other regions are required to provide additional data.

## Conflict of interest

The authors declare that they have no competing interests relevant to this article.
